# A short insertion mutation disrupts genesis of miR-16 and causes increased body weight in domesticated chicken

**DOI:** 10.1038/srep36433

**Published:** 2016-11-03

**Authors:** Xinzheng Jia, Huiran Lin, Qinghua Nie, Xiquan Zhang, Susan J. Lamont

**Affiliations:** 1 Department of Animal Genetics, Breeding and Reproduction, College of Animal Science, South China Agricultural University, Guangzhou 510642, Guangdong, China; 2 Guangdong Provincial Key Lab of Agro-Animal Genomics and Molecular Breeding, and Key Laboratory of Chicken Genetics, Breeding and Reproduction, Ministry of Agriculture, Guangzhou 510642, Guangdong, China; 3 Department of Animal Science, Iowa State University, Ames, IA 50011, USA

## Abstract

Body weight is one of the most important quantitative traits with high heritability in chicken. We previously mapped a quantitative trait locus (QTL) for body weight by genome-wide association study (GWAS) in an F2 chicken resource population. To identify the causal mutations linked to this QTL, expression profiles were determined on livers of high-weight and low-weight chicken lines by microarray. Combining the expression pattern with SNP effects by GWAS, miR-16 was identified as the most likely potential candidate with a 3.8-fold decrease in high-weight lines. Re-sequencing revealed that a 54-bp insertion mutation in the upstream region of miR-15a-16 displayed high allele frequencies in high-weight commercial broiler line. This mutation resulted in lower miR-16 expression by introducing three novel splicing sites instead of the missing 5′ terminal splicing of mature miR-16. Elevating miR-16 significantly inhibited DF-1 chicken embryo cell proliferation, consistent with a role in suppression of cellular growth. The 54-bp insertion was significantly associated with increased body weight, bone size and muscle mass. Also, the insertion mutation tended towards fixation in commercial broilers (*Fst* > 0.4). Our findings revealed a novel causative mutation for body weight regulation that aids our basic understanding of growth regulation in birds.

Body weight, an important trait in all animals, directly reflects the balance of nutrient intake and expenditure, resulting in lean (protein) or fat deposition as well as skeletal growth. Liver, as the main metabolic organ, plays critical roles in regulating body weight through maintaining whole-body energy balance. It acts as a hub to control synthesis and metabolism of molecules such as carbohydrate, fat and protein, which are utilized in various tissues to support whole-body homeostasis^1^. For example, the liver maintains glucose concentrations within a normal range during growth and development by a complex, well-regulated system of enzymes and kinases^2^. Recent studies revealed a skeletal muscle-liver-fat signaling axis that involved in glucose-alanine cycle in mice^3^. When the liver produced fat through *de novo* lipogenesis, a phospholipid released in liver was sent to skeletal muscle cells to burn fat through fatty acid oxidation^4^. Thus, liver is a central organ involved in both fat and muscle deposition, making it an ideal tissue to explore the genetic regulation of body weight and composition.

Chicken, one of the most economically important food-producing animals, provides an excellent example of genetic control of body weight. After 60 years of intensive genetic selection for growth, modern commercial meat-type broilers are three-fold heavier than random control broilers^5^. Recently, advanced genotyping technologies make it possible to characterize genetic variations in large populations and identify loci associated with complex traits at genome-wide level. Through genome resequencing, more than 7,000,000 single nucleotide polymorphisms (SNP) were identified across domestic and wild chickens^6^. To date, high density SNP panels (60 K and 600 K SNP-Chip) have been successfully used to study distinct natural and experimental chicken populations, resulting in identification of many quantitative trait loci (QTL) and candidate genes for body weight^7^,^8^,^9^. In our previous study, using a 60 K SNP-Chip and an intercross of indigenous chickens and commercial broilers, a 3 Mb region on chromosome 1 was identified with a strong association with body weight^10^. Genome-wide association studies (GWAS) identify variants are likely linked to the causal mutations, but do not functionally identify the causal mutations themselves^11^. To identify causal variants requires high-resolution recombination or linkage disequilibrium mapping to nominate putative candidate genes, followed by functional investigation and gene expression analyses^12^. It remains a challenging task to search for the causal mutations linked to observed QTLs responsible for complex biologic traits.

The new strategy of analyzing expression of genes within QTL regions would help to narrow down the numbers of candidate genes or causal mutations responsible for body weight or other complex traits. Using this method, a splice site mutation was identified in pig to cause poor meat quality by affecting expression levels of the PHKG1 gene^13^. Previously, we mapped QTL for body weight in an F2 resource population produced by crossing commercial and indigenous chickens^10^. In the current study we analyzed expression profiles of candidate genes within this QTL region on chromosome 1 (167 to 170 Mb) with the objective to determine the causal mutations contributing to body weight regulation. The results revealed that a 54-bp insertion in the upstream region of miR-15a-16decreased expression of miR-16, resulting in significantly increased body weight gain.

## Results

### Integrated analysis of QTLs with liver transcriptome supported miR-16-1 as a major candidate

**Figure 1:**
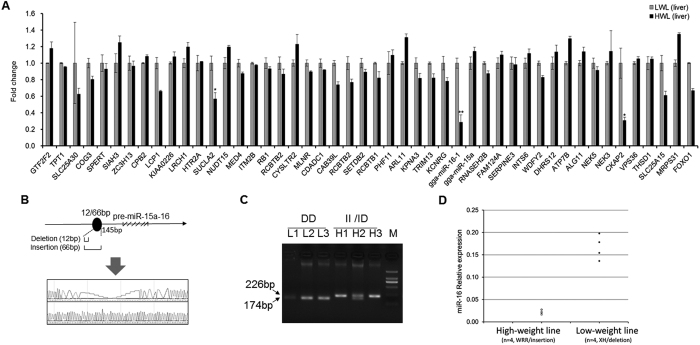
Identification of miR-16-1 as the major candidate gene. (**A**) Gene expression profiling of all the candidate genes within the QTL region mapped by genome-wide association study (GWAS) on body weight. Microarray was performed for mRNA and miRNA expression in liver tissue from high-weight and low-weight line chickens. Differentially expressed mRNAs and miRNAs were identified by ANOVA analysis and false-positive (Fold change >1.5; *p* < 0.01). (**B**) A 54-bp insertion mutation was identified in the primary of miR-15a-16 in chickens. This mutation was located 145 bp upstream of miR-15a-16 precursor. (**C**) Gel electrophoresis was performed for mutation detection in the distinct chicken lines. (**D**) miR-16 expression in the muscle tissue of fast-growing and slow-growing female chickens (n = 4 per line). The fast-growing line is from the white recessive rock breed with insertion homozygous genotype of the miR-15a-16. The slow-growing line is the Xinghua indigenous breed with a wild deleltion homozygous genotype of miR-15a-16. Relative expression values were calculated using the 2^−∆Ct^ method. The data are presented as the means ± SD. *p < 0.05 and **p < 0.01 were estimated by Student’s t-test.

In our previous studies, a GWAS was performed to determine the genetic architecture of body weight in an F2 intercross (of a fast-growing and a slow-growing line), resulting in a 3 Mb QTL region on Chromosome 1, which contains nearly all the significant SNP effects on body weight^10^. This QTL region was also confirmed to be significantly associated with body weight in an independent study^8^. To dissect the causative mutation within this major QTL region, expression profiling of 62 coding genes and 2 miRNAs mapping to this interval was conducted using livers of fast-growing and slow-growing birds. Forty-six genes and miR-15a/16 were successfully detected (Fig. 1A), of which three genes (SUCLA2, CKAP2 and miR-16) exhibited significantly decreased expression in the high weight lines. Integrating with GWAS results, only miR-16 locates nearby two of the most significantly associated loci (rs14916980 and rs13972116; Supplementary Table S1), which reached genome-wide significance on chicken growth^8^. Also, miR-16, the most differentially expressed gene with 3.4-fold down-regulation, plays a critical role in organ growth and development. miR-16 was previously reported to mediate several essential growth-related signaling pathways including Nodal signaling, PI3K/AKT signaling, MAPK signaling and FGF signaling^14^,^15^,^16^. Therefore, it is reasonable to propose that miR-16-1 was the regulator responsible for the control of growth.

### A 54-bp insertion was correlated with miR-16 expression

To search for potentially causal mutations affecting miR-16 expression, we focused on detecting variations in flanking regions of the miR-15a-16 cluster in F0 individuals of Xinghua & White Recessive Rock intercross lines. Three variations were found in the region of miR-15a-16 (Supplementary Table S2). Of note, a 54-bp insertion mutation, located at 145 bp upstream of miR-15a-16 precursor, resulted in changed RNA structure with an added hairpin loop (Fig. 1B). The frequencies of this insertion mutation were 10.7% and 63.3% in low-weight (n = 14) and high-weight groups (n = 14), respectively (Fig. 1C, Supplementary Table S3). Also, no homozygous deletion type was detected in the high-weight line, which suggests that this mutation was potentially undergoing selection for growth rate.

Expression analysis revealed that miR-16 expression in the muscle tissue of birds with the homozygous deletion type (low-weight line, n = 4) was much higher than that in the homozygous insertion type (high-weight line, n = 4) (Fig. 1D), while miR-15a showed no significant change (not show), which is consistent with our liver transcriptomic data. The result is of interest that this short insertion mutation is significantly correlated with miR-16 decreased expression in chicken liver and muscle tissues.

### Characterization of primary transcript of the miR-15a-16-1 cluster

**Figure 2:**
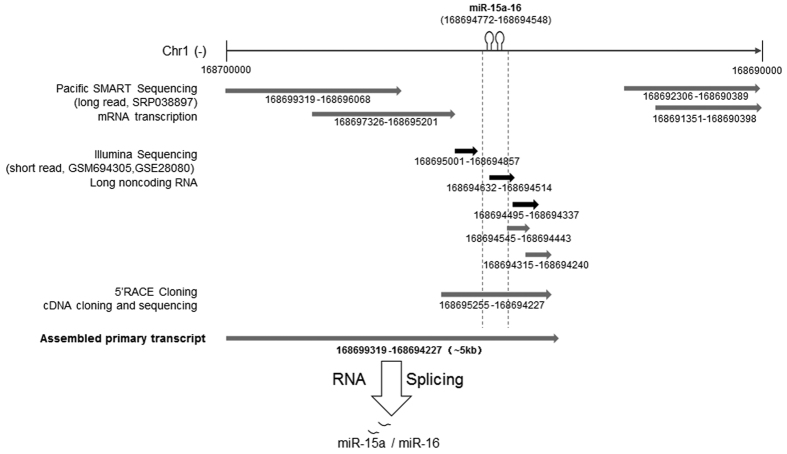
The whole primary sequence of miR-15a-16 was assembled by multiple datasets and SMART cloning sequencing. Integrated analysis of a long noncoding RNA-seq dataset (GSM694305) in muscle, another long noncoding RNA-seq dataset (GSE28080) in muscle and transcriptome data (SRP038897) using long-read sequencing technology resulted in 9 contigs mapped to the flanking of miR-15a-16 cluster. Then, using 5′ rapid amplification of cDNA ends (RACE) technology, a 1028-bp transcript was identified to overlap 6 contigs from high-throughput sequencing, resulting in a transcript isoform fragment of about 5-kb in length.

To determine the impact of mutation on miR-16 biogenesis, it is necessary to characterize the transcription process of miR-15a-16 cluster. High-throughput sequencing and SMART-cloning technologies provided an effective approach to identify potential primary transcripts. Integrated analysis of our previous long noncoding RNA-seq data (GSM694305) in muscle, other long noncoding RNA-seq data (GSE28080) in muscle^17^ and transcriptome data (SRP038897) using long-read sequencing technology^18^ resulted in 9 contigs mapped to the flanking region of the miR-15a-16 cluster (Fig. 2). Then, using 5′ rapid amplification of cDNA ends (RACE) technology, a 1028-bp transcript was identified to overlap 6 contigs from high-throughput sequencing, resulting in a transcript isoform fragment of about 5-kb (Fig. 2). These data revealed that miR-16-1 has an independent transcriptional unit with more than 5-kb of primary RNA transcript.

### 54-bp insertion induces alternative splicing resulting in decreased miR-16 expression

**Figure 3:**
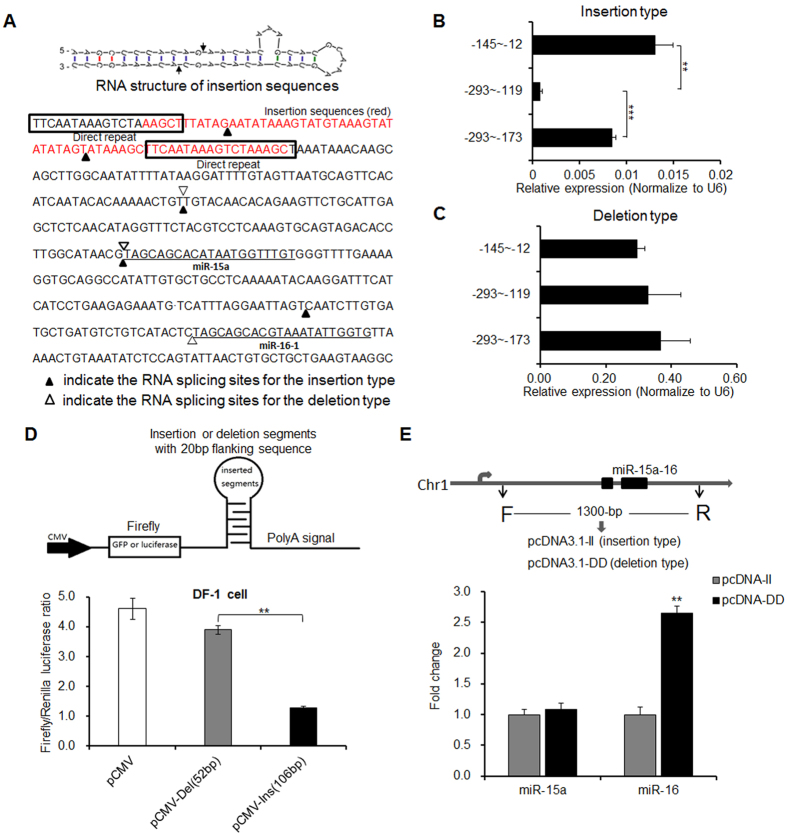
54-bp insertion changed RNA alternative splicing patterns. (**A**) RNA structure of insertion and varied alternative splicing sites compared between insertion and deletion types. (**B**) RNA expression levels of fragments near the insertion region were tested in the birds with the insertion genotype. (**C**) RNA expression levels of fragments near the insertion region were tested in the birds with the deletion genotype. (**D**) Alternative splicing in the insertion region was performed by using a reporter gene system. A 106-bp fragment including the stem-loop insertion, or normal 52-bp fragments, were cloned into the 3′UTR of the reporter gene (luciferase) followed with polyA signals. The recombined vectors were co-transfected with pRL-TK control vector into DF-1 chicken embryo fibroblast cells for 36 h to detect the firefly luciferase activity. (**E**) The effect of insertion mutation on mature miRNA expression *in vitro*. A 1300-bp fragment of the primary transcript of miR-15a-16 was cloned into the pcDNA3.1 vector (pcDNA-II and pcDNA-DD) to overexpress miRNA in DF-1 chicken embryo fibroblast cells. Mature miRNAs were tested by q-PCR. In all panels, data are presented as mean ± SD. **p < 0.01 was estimated by Student’s t-test (n = 3).

Although miR-15a and miR-16-1 are produced from the same primary cluster, the expression levels vary. To confirm whether the insertion mutation alters the alternative splicing pattern during miRNA genesis, 3′RACE PCR was conducted from skeletal muscle RNA of birds with the 54-bp insertion or deletion (wild type). For each cDNA library, 100 clones were randomly selected for sequencing. Five types of alternative splicing sites were detected in the insertion individuals without 5′ terminal splicing of mature miR-16, while 3 types of normal alternative splicing sites were detected in the 5′ terminal of mature miR-15 and miR-16 for the deletion individuals (Fig. 3A; Supplementary Table S4). Interestingly, 2 splicing sites were located within the 54-bp insertion region. Further analyzing the structure of the insertion sequences, there was a typical stem-loop flanked by two 19-bp direct repeats (Fig. 3A). The nearby splicing sites in both stems of the hair-pin structure might therefore be sensitive to recognition by RNase III, a phenomenon that warrants further study.

To validate that alternative splicing events were induced by the insertion mutation, transcriptional expression level of segments in distinct regions flanking the inserted site were quantitated. Only the fragment containing the insertion region (−199 to −293-bp upstream of miRNA precursor) was much lower in expression than the flanking fragments, suggesting that one alternative splicing occurred in the insertion region (−145 to −175-bp) (Fig. 3B,C). The 106-bp fragment containing the insertion mutation and the 52-bp wild fragment were each cloned into the 3′UTR of the firefly luciferase reporter gene followed with polyA signals. After co-transfecting with pRL-TK control vector in DF-1 chicken embryo fibroblast cells, the firefly luciferase activity of insertion mutation type was decreased comparing to the wild type (Fig. 3D), which indicated that the luciferase gene expression was suppressed by the insertion segment in the 3′UTR. Therefore, the insertion segment underwent alternative splicing leading to mRNA instability and lower luciferase activity.

To determine whether alternative splicing induced by the insertion mutation impacted mature miRNA expression, 1300-bp of the primary transcript of miR-15a-16 was cloned into the pcDNA3.1 vector (pcDNA-II and pcDNA-DD), which is used for miRNA over-expression *in vitro*^19^. After transfection in DF-1 chicken embryo fibroblast cells and 36 h of culture, the mature miR-16 expression in the insertion types was significantly lower than that in the deletion types (*p* < *0*.*01*), while mature miR-15a expression exhibited little changes (Fig. 3E). As expected, these observations were generally consistent with our previous results that the 54-bp insertion introduced two novel alternative splicing sites in the mutation region but few splicing sites were detected in the miR-16 5′ terminus. Collectively, these results confirmed that the insertion mutation in the primary transcript induced a special alternative splicing pattern and decreased mature miR-16 expression.

### miR-16 inhibits DF-1 chicken embryo fibroblast cell proliferation

**Figure 4:**
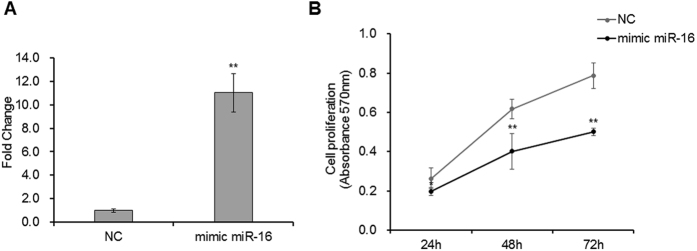
miR-16 suppressed embryonic fibroblast proliferation. (**A**) Overexpression of miR-16 in DF-1 chicken embryo fibroblast cells using mimic miRNA. 20 nM mimics miR-16 and negative random RNA were transfected into cell lines, separately. After 36 h post transfection, the cells were harvested for detecting gene expression (n = 3). U6 was used as the reference gene. Fold change values were calculated using the 2^−∆∆Ct^ method. (**B**) The affection of miR-16 on DF-1 chicken embryo fibroblast cell growth. 20 nM mimic miR-16 and negative random RNA were transfected in DF-1 chicken embryo fibroblast cells, respectively (n = 6). After different periods of culturing (24 h, 48 h and 72 h), the ability of cell proliferation was assayed with MTT assay. The proliferation curves were calculated values of optical density at 570 nm. The curves show that miR-16 significantly inhibited DF-1 chicken embryo fibroblast cell proliferation. The data are presented as the means ± SD. *p < 0.05 and **p < 0.01 were estimated by Student’s t-test.

The miR-16 family induces cell cycle arrest in most cancer cells by regulating multiple cell cycle genes^20^. However, no study has yet addressed the impacts of miR-16 on embryo growth. To verify this, mimic miR-16 was used to up-regulate the expression of miR-16 in DF-1 chicken embryo fibroblast cells (Fig. 4A). The MTT assay showed that miR-16 significantly inhibited DF-1 chicken embryo fibroblast cell proliferation and this effect was constant until 72 h (Fig. 4B).

### The indel mutation was strongly associated with growth traits

**Figure 5:**
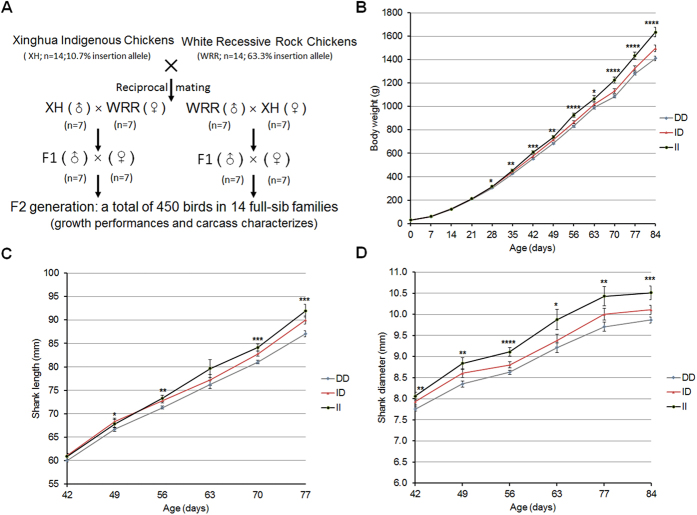
Genetic effect of indel mutation. (**A**) The F2 resource population was constructed by reciprocal intercrosses between Xinghua indigenous chickens and White Rock chickens. A total of 450 birds were measured for growth performance and carcass characteristics. (**B**) Association analysis between different genotypes (DD, homozygous deletion type; ID, heterozygous type; II, homozygous insertion type) and chicken body weight in the F2 designed resource population (XH & WRR). (**C**) Association analysis between different genotypes (DD, ID or II) and chicken shank length in the F2 designed resource population (XH & WRR). (**D**) Association analysis between different genotypes (DD, ID or II) and chicken shank diameter in the F2 designed resource population (XH & WRR). The numbers of DD, ID and II birds were at least 270, 90 and 55, respectively. The data are presented as Least squares mean (LSM) ± Stand error (SE); *p < 0.05, **p < 0.01, ***p < 0.001, ****p < 0.0001.

Artificial selection has produced large phenotypic changes between breeds during domestication. Here, an F2 resource population was constructed by intercrossing an indigenous breed (XH, 10% insertion allele) and commercial broilers (WRR, 63% insertion allele) to carry out a marker-trait association analysis of the 54-bp insertion on growth performance (Fig. 5A). The results revealed a highly significant association between the 54-bp insertion mutation and body weight, shank diameter, and shank length. From 28 to 84 days, body weight of homozygous insertion-type birds (II genotypes) was significantly (*p* < *0*.*01*) heavier than homozygous deletion-type birds (DD genotypes), and at 84-day-old age, the weight gain difference was 224 g (Fig. 5B). The shank diameter and shank length were also larger in the birds containing the 54-bp insertion. From 49 to 84 days, the shank diameters of insertion-type birds were significantly larger than that of deletion-type birds (Fig. 5C). Similarly, the shank lengths were longer in the insertion-type birds (Fig. 5D). These data indicate that the insertion mutation was associated with growth performance, contributing to weight gain and bone growth.

In chicken, growth is highly correlated with carcass traits^21^, which impacts economic value. Here, we showed that body composition traits of carcass weight, semi-eviscerated weight, eviscerated weight, breast muscle weight, leg muscle weight and feathers weight were all significantly associated with the insertion mutation (*p* < *0*.*001*) (Table 1).

### Allele frequency of insertion mutation among distinct chicken populations

Allelic frequency is a reflection of genetic diversity among populations, potentially indicating genetic drift or novel mutation introduction. To help clarify when this insertion mutation occurred during chicken domestication, 12 distinct breeds were used to estimate its allele frequency. Large differences in allelic frequency were found across indigenous breeds, wild birds and commercial breeds. The Red Jungle fowl, the putative ancestor of the domestic chicken, bore the reference genome without the insertion allele. A feral breed from Kauai Island, which represents a breed without recent artificial selection, also exhibited a low allele frequency of the insertion at 6.3%. For the indigenous breeds, which have undergone unsystematic selection and have slow growth, the frequency of insertion allele was about 5% to 21%. However, the insertion allele frequency of two commercial broiler breeds with fast growth were up to 70% (Table 2). According to alignment analysis of reference genomes of other avian species (duck, goose and turkey), no insertion mutation occurred in these species. These results suggest that this short insertion mutation arose early in chicken domestication and responded strongly to recent intense selection for rapid growth and high body weight.

### Differential selection of insertion allele in modern broilers

Positive selection causes spreading and concentrating of advantageous alleles across populations, resulting in a reduction of diversity at the selected loci and potentially, fixation in some populations. To identify the evidence of differential selection in miR-15a-16 during domestication in chickens, pairwise *Fst* values were calculated across 12 chicken populations. The results indicate that a significant selection signature was detected between commercial broilers and other lines. Between these two broiler lines, there was little differentiation. Extremely high differentiation value (more than 0.5) was found between wild chickens and broiler lines, suggesting that the insertion mutation was highly selected in the modern meat-type chickens. Also, most of the indigenous breeds and these commercial lines had significant differentiation signatures (*Fst* > *0*.*4*). In contrast, *Fst* values among the indigenous lines were nearly less than 0.1, indicating no obvious differentiation (Table 3).

## Discussion

Body weight is one of most highly heritable traits, with about 0.24~0.47% heritability at different age in chicken^22^. In this study, we characterized a causal mutation affecting body weight in the chicken. We integrated our previous QTL mapping for body weight by GWAS with expression profiling of liver to reduce the number of candidate genes potentially linked to causative mutations. Our results supported miR-16 as a highly likely candidate gene, based upon location in the major QTL region by GWAS, and significantly different expression in liver and muscle tissue of fast- versus slow-growing chickens. Elevating miR-16 expression also suppressed DF-1 chicken embryo fibroblast cell growth. A 54-bp insertion mutation in the upstream of precusor was identified to be a causative mutation that disrupted miR-16 genesis and contributed to weight gain during most of the growth phase. Several independent observations support the 54-bp insertion as the causal mutation: (1) miR-15a-16 is a large (more thab 5-kb)independent transcript, which suggests that miR-15a-16 would be regulated by multiple transcription factors and alternative splicing. (2) The 54-bp insertion mutation changed the miR-16 alternative splicing pattern. Alternative isoform analysis demonstrated that the insertion mutation introduced three novel alternative splicing sites instead of 5′ terminal splicing site of mature miR-16. Also two sites were within the insertion region. (3) The 54-bp nsertion mutation caused lower miR-16 expression. In both *in vivo* tissue and *in vitro* cell experiments, the insertion type resulted in lower abundance of mature miR-16. (4) The insertion mutation was associated with high body weight and growth performance. In the F2 population, this mutation was significantly associated with most growth traits such as body weight, bone size and muscle mass (*p* < *0*.*01*). (5) The insertion mutation underwent selection during broiler commercial selection. Allele frequency analysis showed that the insertion allele tended toward fixation in the modern commercial chicken populations but not in indigenous and wild ones (*Fst* > *0*.*4*). Take together, our findings revealed a novel causal mutation in miR-16 contributing to genetic regulation of body weight.

Through analysis of liver transcriptome differencess between high- and low-weight lines, we reduced the number of candidate genes locatedin the QTL region to three major candidates that showed differential expression, SUCLA2, CKAP2 and miR-16. According to functional annotation, SUCLA2 is a mitochondrial enzyme that catalyzes the reverse reaction of succinyl-CoA and ADP or GDP to succinate and ATP or GTP^23^. Deletion or mutation of SUCLA2 leads to elevate methylmalonic acid, resulting in a serious of complex phenotypes comprising muscle atrophy, hyperkinesia, severe hearing impairment and postnatal growth retardation^24^. CKAP2 is a microtubule-associated protein that is critical for the normal mitotic progression^25^. Although both SUCLA2 and CKAP2 were reported to be related to growth regulation, none of the SNPs located in them reached genome-wide significance on chicken growth. For miR-16-1, the two loci of rs13972116 and rs14916980, located up- and down-stream of the precursor showed a large effect on chicken growth^8^. Thus, we hypothesized that miR-16 should be a causal gene regulating chicken growth. We identified a 54-bp insertion close to miR-16 as a causal mutation that likely impacts body weight gain by disrupting miR-16 expression.

Although no direct relationship between miR-16 and body weight has been previously reported, miR-16 was proven to play a critical role in regulating organ growth and development through mediating several essential growth-related signaling pathways including Nodal signaling, PI3K/AKT signaling, MAPK signaling and FGF signaling^14^,^15^,^16^. *In vitro*, miR-16 induced G1 arrest in A549 cells by targeting multiple cell cycle genes such as CCND1, CCND3 and CCNE1^20^. Our results were consistent with previous reports, in that our study also indicated that miR-16 impacted chicken embryonic development by significantly inhibiting DF-1 chicken embryo fibroblast cell proliferation. Additionally, miR-16 promotes cell apoptosis via regulating the Bcl2 gene in tumors and liver disease^26^,^27^. Collectively, these data suggest that miR-16 might affect body growth by repressing growth of various cell types and inducing apoptosis.

Artificial selection shaped the amount and distribution of genetic variation during the domestication process, and also in the recent development of breeds and commercial production populations, especially in poulty. As a result, many beneficial alleles were fixed or nearly fixed in specificl breeds as genetic changes occurred in population differentiation accompanying the domestication preocess^28^. In our study, the 54-bp insertion allele frequencies in the ancestor, indigenous and modern chickens displayed significant population differentiation. Commercial broilers had the highest allele frequency, up to 70%, while the ancient Red Jungle fowl was zero and indigenous breeds were only 6–20%. The insertion allele frequency in feral chickens from Kauai island was 6.3% (n = 24) with little difference from Red Jungle fowl. This was consistent with a previous report that Kauai chickens showed a mixure of domestic chickens and ancient Red Jungle fowl during the last few decades^29^. Fst statistical analysis also demostrated that the growth-advantagious allele of the miR-16 insertion mutation was approaching fixation in the fast-growing commercial lines (*Fst* > *0*.*4*) during recent artificial selection after long-term domestication.

The 54-bp insertion has not previously been reported in selection sweep or re-sequencing studies of chickens. Through de-novo alignment of insertion fragements to these reported genome data from distinct populations, we found that this insertion mutation does exist in multiple populations, for example in Kauai’s feral chickens^29^. In an extensive study on whole genome selection during chicken domestication, 1,300 deletions and several putative selective sweeps were detected in eight different populations of domestic chickens as well as red jungle fowl, and the strongest selective sweeps were found to contain the TSHR and SH3RF2 genes^6^. However, using the technology of 36-bp sequencing reads, only 100 bp or larger indel mutations could be reliably detected. For short insertion or deletion mutations (40–100 bp) in the genome, it is hard to test using the current SNP-panel or re-sequencing methods, due to the short reads and assembly strategy^30^. The better future strategy for indel detection is to combine deep sequencing with long-read traditional sequcing or single molecular sequencing and de-novo assembly.

In summary, through co-localization of a QTL for body weight identified by GWAS and elements differentially expressed in liver, we have fine mapped miR-16 as the major candidate gene likely linked to a causal mutation for several growth and body composition traits. A 54-bp insertion mutation in 145-bp upstream of precursor miR-15a-16 was identified as the causal mutation, resulting in increased body weight, bone size and muscle mass by disrupting alternative splicing during miR-16 biogenesis. Moreover, allele frequency analysis showed significant differences in distinct chicken populations during domestication, tending toward fixation in commercial broilers (*Fst* > 0.4). In total, our findings provided new insights to help understand body weight regulation and provides a biomarker that may contribute to improving genetic selection for enhanced performance in poultry.

## Methods

### Ethics statement

All the experimental protocols in this study met the guidelines of the Animal Care and Use Committee of the South China Agricultural University (SCAU) (Guangzhou, People’s Republic of China). All animal experiments of this study were approved by the Animal Care and Use Committee of the SCAU with approval number SCAU#0011.

### Animals

A total of 12 populations were used for the allele frequency analysis of the 54-bp indel in the 5′ region of pre-miR-15a-16, including 2 wild breeds (Kauai feral chickens and Red Junglefowl), 8 indigenous chickens (Xinghua chicken, Wenchang chicken, Gushi chicken, Naked-neck chicken, Qingyuan chicken (QY), Fighting chicken, dwarf yellow chicken and Tibetan chicken) and 2 commercial breeds (White Rock chicken #1 and #2).

An F2 designed family population was constructed for genetic association analysis. Fourteen Xinghua indigenous chickens (XH; a slow-growing breed containing 10.71% insertion alleles) and 14 White Recessive Rock chickens (WRR, a fast-growing breed containing 63.33% insertion alleles) were intercrossed to generate a F2 resource population by reciprocal mating. A total of 450 birds in 14 full-sib families were raised in floor pens on corn–soybean-based diets meeting all NRC requirements. Detailed growths and carcass traits were measured and recorded after hatching (day 1–90) and slaughter at 90-days old.

Liver tissues from 3 indigenous QY and 3 commercial WRR chicken at about 7-week-old were used for miRNA microarray analysis. Another 8 birds from XH and WRR (n = 4 per population) were used to confirm miR-16 expression pattern. In our previous study, liver tissues from 3 indigenous XH and 3 commercial WRR chicken at about 7-weeks-old were used for mRNA microarray analysis^31^. QY and XH chicken are populations with similar growth performance, and thus used to represent populations with the same general growth characteristics and to contrast with fast-growing WRR.

### Cell culture

A chicken embryo fibroblast (DF-1) cell line was obtained from the Cell Bank of Committee on Type Culture Collection of the Chinese Academy of Sciences. It was cultured in DMEM (Invitrogen) with 10% fetal bovine serum (FBS) (Gibco) and 0.2% penicillin/streptomycin (Invitrogen) at 37 °C with 5% CO_2_.

### DNA and RNA isolation

The DNA samples were isolated from chicken blood containing EDTA following the standard phenol/chloroform method. Total RNA was isolated from liver and muscle using Trizol (Invitrogen) according to the manufacturer’s protocol. RNA quality and quantity were tested by using the Agilent 2100 Bioanalyzer system according to Agilent RNA 6000 Nano Kit (Agilent) and Nanodrop2000 manufacturers’ instructions.

### Microarray analysis

Six liver tissues from QY and WRR (n = 3 from each line) were analyzed on the GeneChip miRNA Array (Affymetrix). For each sample, 1 μg of total RNA was labeled using FlashTag Biotin RNA Labelling Kit (Genisphere). Array staining, washing and scanning were conducted according to standard Affymetrix protocols using the GeneChip Scanner 3000. Raw data were analyzed with the Partek Genomics Suite 6.4 (www.partek.com) and normalized using the Robust Multichip Analyses (RMA) method. After data normalization, significance was determined by ANOVA analysis and false-positive reduction (*p* < 0.01). mRNA expression was based on our previous microarray data^31^. The microarray dataset was deposited to Gene Expression Omnibus (GEO) under accession number GSE80580.

### Real-time PCR

To determine the relationship between the mutation and miRNA expression level, real time PCR was performed to detect mature miR-15a and miR-16. The miScript Reverse Transcription kit (Qiagen) and miScript SYBR Green PCR kit (Qiagen) were used for cDNA synthesis and quantity PCR detection, respectively. The procedures were conducted according to the manufacturer’s protocols. U6 was used to normalize the relative expression. Primer information is provided in supplementary Table S5. The relative expression of miRNA was calculated using 2^−∆Ct^ or comparative 2^−∆∆Ct^ methods^32^, the latter of which was used to estimate the expression fold change between two different samples.

### Genetic variation and genotyping

Direct PCR sequencing was performed to identify variations of primary of miR-15a-16 in 20 birds of XH and WRR lines. To gain additional phenotype information, 12 different populations were used to test allele frequency of the short insertion mutation by PCR amplification and gel detection. The related primers were provided in the supplementary Table S5. We utilized high-throughput sequencing datasets available in the NCBI database (http://www.ncbi.nlm.nih.gov/) to explore the genotype information of miR-15a-16 in chickens from around the world. Blast-2.3.0 (ftp.ncbi.nlm.nih.gov/blast/executables/LATEST) was used to align the cleaned reads to complementary sequences of insertion and deletion alleles (100-bp including the indel region).

### MiR-16 transcription and alternative splicing

To determine the primary sequence of miR-15a-16, we integrated high-throughput sequencing data (GSM694305, SRP038897 and GSE28080) from the NCBI SRA database (www.ncbi.nlm.nih.gov/sra) and 5′ RACE cloning sequences. Blast-2.3.0 (ftp.ncbi.nlm.nih.gov/blast/executables/LATEST) was used to align the cleaned reads to complementary sequence of Chr 1: 168.69–168.70 Mb. 5′ RACE and 3′ RACE PCR were performed by using the SMART RACE cDNA Amplification kit (Takara) following the manufacturer’s instructions. PCR products were cloned into pMD-18T (Takara) and sequenced by Invitrogen Co. Ltd (Guangzhou, China). Insertion or deletion segments with 20 bp flanking sequence were cloned into the reporter vector pCMV-Firefly-3′UTR to test alternative splicing in the insertion segments. The recombined vectors were transfected with pRL-TK vectors into DF-1 chicken embryo fibroblast cells, separately. After 36 h post transfection, cells were harvested to detect luciferase activity using the Dual-Luciferase Reporter Assay System (E1910) according to the manufacturer’s instructions (Promega). Renilla luciferase activity served as the normalization control. If the splicing event occurred, the stability of firefly gene mRNA was destroyed resulting in reduced luciferase activity. To investigate the impacts of insertion mutation on mature miR-16, fragments of about 1300 bp in length including the miR-15a-16 precursor and mutation region were constructed into pcDNA3.1(+). Finally, two vectors including pcDNA3.1 insertion vector and pcDNA3.1-deletion vector were transfected into DF-1 chicken embryo fibroblast cells to analyze mature miRNA expression levels. The RNA secondary structure was formed by Mfold soft systems (http://unafold.rna.albany.edu/). Primer information is provided in supplementary Table S5.

### MTT assay

Mimic miRNA (Qiagen) were used to overexpress miR-16, with negative random RNA mimic as a control. The MTT proliferation assay kit (Invitrogen) was used to test cell proliferation effects of miR-16, which was conducted by 6 replications for each group. In brief, 5 × 10^4^ cells per well were seeded in 48-well plates and incubated for 24 h, and then 20 nM mimics were transfected into the cell lines with measurements made every 24 h according to the manufacturer’s protocol of MTT proliferation assay kit (Invitrogen). The proliferation curves were calculated values of optical density at 570 nm.

### Population differentiation

The fixation index of different populations (*Fst*) was estimated to characterize the population differentiation among indigenous, wild and commercial breeds by using GENEPOP 4.5 programs^33^.

### Association analyses of the 54-bp indel mutation with phenotypic traits

Marker-trait association analyses were performed by the SAS V8 (SAS Institute, 1996) and the genetic effects were analyzed using the following GLM model:


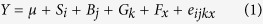


where Y is a trait observation, μ is the overall population mean, S is the fixed effect of sex (male or female), B is the fixed effect of hatch, G is the fixed effect of genotype, F is random effect of dam and e is the residual random error. Multiple comparisons were performed to calculate the least square means (LSM) by the Duncan’s Multiple Range test. Details are reported in our previous study^34^.

### Statistical analysis

The significance of the differences between contrasted groups was calculated by an unpaired Student T-test. *p* < 0.05 and *p* < 0.01 were considered significant and highly significant, respectively.

## Additional Information

**How to cite this article**: Jia, X. *et al*. A short insertion mutation disrupts genesis of miR-16 and causes increased body weight in domesticated chicken. *Sci. Rep.* **6**, 36433; doi: 10.1038/srep36433 (2016).

**Publisher’s note:** Springer Nature remains neutral with regard to jurisdictional claims in published maps and institutional affiliations.

## Supplementary Material

Supplementary Information

## Figures and Tables

**Table 1:** The short insertion mutation was associated with carcass traits.

Traits	Genotype			P value
	DD	ID	II	
Carcass weight	1292.81 ± 14.49 (282)	1366.99 ± 21.99 (99)	1399.27 ± 29.63 (55)	0.0004
Semi-eviscerated weight	1180.75 ± 13.75 (282)	1252.57 ± 20.58 (99)	1278.36 ± 27.74 (55)	0.0003
Eviscerated weight	1023.85 ± 12.14 (282)	1086.07 ± 18.17 (99)	1108.54 ± 24.49 (55)	0.0004
Breast muscle weight	88.17 ± 1.1 (282)	93.58 ± 1.67 (99)	95.56 ± 2.25 (55)	0.0009
Leg muscle weight	112.75 ± 1.44 (282)	119.22 ± 2.18 (99)	120.9 ± 2.94 (55)	0.0044
Feathers weight	62.87 ± 0.73 (282)	66.62 ± 1.1 (99)	68.72 ± 1.49 (55)	0.0001
Subcutaneous fat thickness	4.17 ± 0.09 (282)	4.13 ± 0.13 (99)	3.97 ± 0.18 (55)	0.5816
Abdominal Fat Weight	28.1 ± 1.15 (282)	26.75 ± 1.74 (99)	28.74 ± 2.35 (55)	0.7081

Multiple comparisons were performed to calculate differences in the least square means (LSM) by the Duncan’s Multiple Range test. The numbers of DD (homozygous deletion type), ID (heterozygous type) and II (homozygous insertion type) birds were at least 270, 90 and 55. The data were presented as (LSM) ± Standard error (SE).

**Table 2:** Allele frequency of insertion mutation in miR-15a-16 among wild, indigenous and commercial chickens.

Breeds	Character	Fre (I)	Population	Ref^#^
Kauai feral chicken*	Wild	6.3%	24	29
Red Junglefowl	Ancestor Wild	0.0%	6	35
Xinghua chicken	meat-egg	14.1%	252	
Wenchang chicken	meat-egg	22.9%	48	
Gushi chicken	meat-egg	7.6%	82	
dwarf yellow chicken	meat-egg	4.6%	65	
Naked-neck chicken	meat-egg	20.5%	39	
Qingyuan chicken	meat-egg	13.2%	72	
Tibetan chicken	meat-egg	10.0%	10	35
Fighting chicken	meat-egg	18.8%	8	35
White Rock chicken 1	broiler	73.5%	81	
White Rock chicken 2	broiler	69.6%	69	

The birds were genotyped by analysis of the genome re-sequencing datasets from SRA database. *Kauai is a Hawaiian island with various types of feral chickens.

**Table 3:** Pairwise fixation index (*Fst*) among wild, indigenous and commercial chickens.

pop	Kauai feral chickens	Red Jungle fowl	Xinghua chicken	Wenchang chicken	Gushi chicken	Dwarf yellow chicken	Naked-neck chicken	Qingyuan chicken	Tibetan chicken	Fighting chicken	White Rock chicken #1
Red Junglefowl	−0.0102										
Xinghua chicken	0.0009	0.0194									
Wenchang chicken	0.0783	0.1067	0.0538								
Gushi chicken	−0.0162	−0.0186	0.0014	0.0831							
Dwarf yellow chicken	−0.0110	−0.0182	0.0187	0.1356	0.0003						
Naked-neck chicken	0.0576	0.0787	0.0323	−0.0109	0.0604	0.1145					
Qingyuan chicken	0.0101	0.0356	−0.0023	0.0247	0.0073	0.0366	0.0093				
Tibetan chicken	−0.0240	0.0195	−0.0266	0.0215	−0.0365	0.0011	−0.0011	−0.0238			
Fighting chicken	0.0526	0.1137	−0.0043	−0.0301	0.0263	0.1263	−0.0448	−0.0207	−0.0188		
White Rock chicken #1	0.5750	0.5879	0.6194	0.3997	0.6138	0.6473	0.4262	0.5331	0.5143	0.4259	
White Rock chicken #2	0.5319	0.5406	0.5871	0.3497	0.5789	0.6150	0.3759	0.4913	0.4629	0.3685	−0.0030

All *Fst* statistical values were tested by GENEPOP 4.5 with Bonferroni correction. The cutoff value was set at *p* < 0.002 at 5% confidence interval.
