# Supplementary material for: A short insertion mutation disrupts genesis of miR-16 and causes increased body weight in domesticated chicken
Source: Sci Rep. 2016 Nov 3;6:36433. doi: 10.1038/srep36433 (PMC5093740; doi:10.1038/srep36433)
Supplement: Supplementary Information [file srep36433-s1.pdf]

A short insertion mutation disrupts genesis of miR-16 and causes increased body weight in domesticated chicken

Xinzheng Jia[1,2,3], Huiran Lin[1,2], Qinghua Nie[1,2]*, Xiquan Zhang[1,2], Susan J. Lamont[3]

[1]Department of Animal Genetics, Breeding and Reproduction, College of Animal Science, South China Agricultural University, Guangzhou 510642, Guangdong, China.

[2]Guangdong Provincial Key Lab of Agro-Animal Genomics and Molecular Breeding, and Key Laboratory of Chicken Genetics, Breeding and Reproduction, Ministry of Agriculture, Guangzhou 510642, Guangdong, China.

[3]Department of Animal Science, Iowa State University, Ames, IA 50011, USA

*To whom correspondence should be addressed. Tel. 86-20-85285759. Fax. 86-20-85280740. Email: nqinghua@scau.edu.cn.

Supplementary data

Supplementary Table S1. Annotation of differentially expressed genes and GWAS data.

Nearby genes	Gene Location	SNP ID	SNP position (galGal4)	Traits with SNP effects* (GWAS)
**SUCLA2**	167942414-167962711	GGaluGA054587	167936820	
		Gga_rs15496102	167956206	
**mIR-16-1**	168694548-168694631	Gga_rs14916980	168692233	SC6, SC8
		Gga_rs15498187	168713010	
		Gga_rs13972116	168745370	BW10, BW12, GR0–4, GR0-8, SL10, SL12, SC 10, SC12, SW, AFW
**CKAP2**	169701518-169710954	GGaluGA055247	169702400	
		Gga_rs13973293	169712027	FCR 6–8

*SNP effects were identified in two independent GWAS of F2 intercross lines of fast-growing and slow-growing birds, conducted by Xie et al. (2012), and Sheng et al. (2013). SC, shank circumference; BW, body weight; GR, growth rate; SL, shank length; SW, stomach weight; AFW, abdominal fat weight; FCR, feed conversion ratio.

Supplementary Table S2. Mutations of miR-15a-16 in XH and WRR chickens. *chicken SNP data bank (www.ncbi.nlm.nih.gov/snp).

Marker	Chr	Chr Position	Type	SNP to refSNP
Indel	1	168694917	54-bp Del/Ins	
SNP1*	1	168694793	C/T	rs315624237
SNP2	1	168694612	C/T	

Supplementary Table S3. Genotype of F0 generation of XH & WRR family population. Gel detection was performed for mutation genotyping in 28 F0 individuals. DD, homozygous deletion type; ID, heterozygous type; II, homozygous insertion type.

	**Low-weigh line**			**High-weight line**		
Genotype	II	ID	DD	II	ID	DD
Number	0	3	11	6	7	1
Frequency(I)	**10.71%**			**63.33%**		

Supplementary Table S4. Different splicing patterns between insertion and deletion types. 3'RACE PCR technology was performed for detecting alternative splicing. For each genotype, total 100 clones were randomly chosen for sequencing.

	**Splicing 1**	**Splicing 2**	**Splicing 3**	**Splicing 4**	**Splicing 5**	**Splicing 6**
Insertion	14% (13)	11% (10)	13% (12)	34% (32)	28% (26)	0% (0)
Deletion	0% (0)	0% (0)	14% (13)	0% (0)	37% (34)	47% (41)

Supplementary Table S5. Primers used for PCR amplification and vector construction in this study.

**Gene**	**Forward primer (5' to 3')**	**Reverse primer (5' to 3')**	**Purpose**
gga-miR-16	tagcagcacgtaaatattggtg	miScript Universal primer	Validation of differentially expressed miRNAs by qPCR
gga-miR-15a	tagcagcacataatggtttgt	miScript Universal primer	
U6	cgatacagagaagattagcatgg	miScript Universal primer	
RACE-5-F	aagcagtggtaacaacgcagagt (NUM)	ttaaagtgatggtgttgttataat	RACE PCR
RACE-3-F	ctgctacaggctacttgctaa	aagcagtggtaacaacgcagagt (NUM)	
Pri-miR-16-1	tgcagttcacatcaatacac	ttaccgaagcactgttagac	Scanning mutation
Pri-miR-16-2	ggaccataacagattcttgat	ggctacaaacctgctgtgctt	

miR-16-1(GT)	tcctcagtaaatacccacata	gaactgcattaactacaaaatc	Genotyping for insertion mutation
miR-(-12-145)	taaataaacaagcagcttggc	tttgaggacgtagaaacctat	qPCR for insertion region
miR-(-293-119)	tgctacaggctacttgctaac	atattgccaagctgcttgtt	
miR-(-293-173)	tgctacaggctacttgctaac	tttagactttattgaaatct	
pcDNA-pri-miR	gacggtaccagggtgatttcatcctacgc	atttgcggccgtagttaacaattttagggtc	Constructing the pcDNA-II and pcDNA-DD

Reference

1. Sheng, Z. *et al.* Genetic dissection of growth traits in a Chinese indigenous x commercial broiler chicken cross. *BMC Genomics* **14**, 151 (2013).

2. Xie, L. *et al.* Genome-wide association study identified a narrow chromosome 1 region associated with chicken growth traits. *PLoS One* **7**, e30910 (2012).
